# Complex Magnetization Harmonics of Polydispersive Magnetic Nanoclusters

**DOI:** 10.3390/nano8060424

**Published:** 2018-06-11

**Authors:** Suko Bagus Trisnanto, Yasushi Takemura

**Affiliations:** Department of Electrical and Computer Engineering, Yokohama National University, Yokohama 240-8501, Japan; suko-trisnanto-zt@ynu.ac.jp

**Keywords:** magnetic nanoclusters, magnetic interactions, magnetization harmonics, polydispersity, magnetic theranostics

## Abstract

Understanding magnetic interparticle interactions within a single hydrodynamic volume of polydispersed magnetic nanoparticles and the resulting nonlinear magnetization properties is critical for their implementation in magnetic theranostics. However, in general, the field-dependent static and dynamic magnetization measurements may only highlight polydispersity effects including magnetic moment and size distributions. Therefore, as a complement to such typical analysis of hysteretic magnetization curves, we spectroscopically examined the complex magnetization harmonics of magnetic nanoclusters either dispersed in a liquid medium or immobilized by a hydrocolloid polymer, later to emphasize the harmonic characteristics for different core sizes. In the case of superparamagnetic nanoclusters with a 4-nm primary size, particularly, we correlated the negative quadrature components of the third-harmonic susceptibility with an insignificant cluster rotation induced by the oscillatory field. Moreover, the field-dependent in-phase components appear to be frequency-independent, suggesting a weak damping effect on the moment dynamics. The characteristic of the Néel time constant further supports this argument by showing a smaller dependence on the applied dc bias field, in comparison to that of larger cores. These findings show that the complex harmonic components of the magnetization are important attributes to the interacting cores of a magnetic nanocluster.

## 1. Introduction

Superparamagnetism of magnetic nanostructures has been one of the main concepts underlining recent theranostic applications [1,2]. This property is well understood as a physical manifestation of the dynamic behavior of a macrospin system above its blocking temperature, which results in a non-hysteretic magnetization curve against a periodically ramped magnetic field at extremely low frequencies [3,4]. For a superparamagnetic nanoparticle system, the corresponding static magnetization easily saturates according to the Langevin model with a high saturation value [4]. Owing to the delays in the oscillatory magnetic-field-induced relaxation responses Néel and Brownian relaxations [5,6], the system may exhibit a ferromagnetic-like behavior by showing magnetization reversal with a finite coercivity [7,8]. The resulting heat dissipation and magnetic signals are therefore promising for magnetic hyperthermia, particle imaging, and biosensing [9,10,11]. Nevertheless, these applications substantially demand a good understanding of colloidal magnetic nanostructures and their nonlinear magnetization dynamics to acquire high hyperthermic efficiency and nonlinear magnetization at low-field regimes.

Specific to the case of magnetic nanoclusters, the dipolar interparticle interaction of the core particles within a single hydrodynamic volume is one of the critical considerations in explaining the nonlinear field-dependent magnetization responses [12,13], in addition to the polydispersity effects (e.g., magnetic moment and size distributions, and the corresponding anisotropy energies). In fact, there is a trade-off in whether such magnetic interaction promotes a negative contribution to the magnetization response at the low-field regime [14]. Therefore, understanding magnetic interaction is critical as it potentially affects the room temperature magnetism due to an increase of the blocking temperature. In this regard, our study focuses on analyzing the intrinsic dipolar magnetism of colloidal magnetic nanoclusters in terms of the complex magnetization harmonics. We hypothesize that the change in the nonlinear magnetization properties attributed to dipole-dipole interactions should correlate with both the real and imaginary parts of the apparent magnetization harmonics. Nevertheless, recent interest in exploring these harmonic signals toward magnetic particle imaging (MPI), for instance, appears to ignore such phase-delay characteristics of the complex magnetization harmonics but emphasize the respective magnitudes instead [15]. Thus, this article also aims to introduce the physical meaning of the complex magnetization harmonics related to the dynamics of magnetic nanoclusters, particularly for the benefit of MPI.

## 2. Materials and Methods

**Figure 1:**
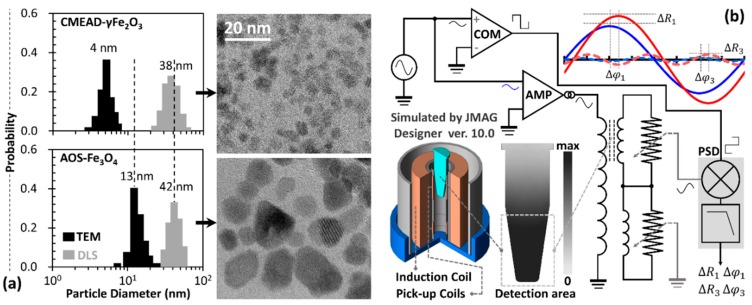
(**a**) Transmission electron microscopy images and dynamic light scattering measurements of the characterized ferrofluid samples; (**b**) Phase-sensitive detection system to measure the complex magnetization harmonics of a 0.1-mL ferrofluid sample placed within an area with a spatially uniform field-distribution. The signal amplitude and phase differences at the fundamental and third-harmonic frequencies ($\Delta R_{1}$, $\Delta \varphi_{1}$, $\Delta R_{3}$, and $\Delta \varphi_{3}$, respectively) were recorded while varying field strength and frequency of the applied fields.

Depending on the interparticle distance and the primary size-dependent magnetic moment of the core particles, magnetic nanoclusters may have a non-zero effective (cluster) moment. The nanoclusters then may undergo a corresponding oscillatory-field induced physical rotation at low frequencies, which is spectroscopically observable from the imaginary part of the magnetic susceptibility [16]. More specifically, the superparamagnetic nanoclusters with very small core-particles may preferably demonstrate moment relaxation, owing to the low anisotropy energy barrier and weak dipolar magnetism [17]. In this regard, we evaluated the complex magnetization harmonics of carboxymethyl-diethylaminoethyl dextran (CMEAD)-coated maghemite nanoparticles (Meito Sangyo Co., Ltd., Kiyosu-shi, Japan) and compared them to those of sodium α-olefin sulfonate (AOS)-coated magnetite nanoparticles (Sigma Hi-Chemical Inc., Chigasaki-shi, Japan, product name: M-300). In terms of physical properties (Figure 1a), these polydispersive ferrofluids contain magnetic nanoclusters with similar secondary sizes. However, the CMEAD-γFe_2_O_3_ core particles with an average primary size of about 4 nm are three times smaller than the AOS-Fe_3_O_4_ core particles. We used a typical phase-sensitive detection method to measure the complex third-harmonic signals (Figure 1b). In addition, we preliminarily characterized the static and dynamic magnetization curves of the ferrofluid samples, as well as the field-dependent relaxation time constant of the immobilized particles. During all measurements, the concentration and sample volume were set to 28 mg-Fe mL^−1^ and 0.1 mL, respectively.

## 3. Results and Discussion

### 3.1. Magnetism of CMEAD-γFe_2_O_3_

**Figure 2:**
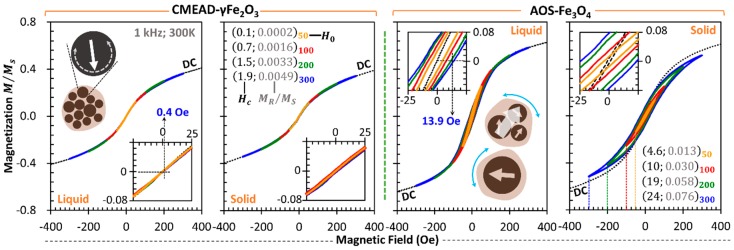
Dynamic magnetization curves of the ferrofluid samples at 1 kHz. The different coercive fields $H_{C}$ and remanences $M_{R}$ within low-field regimes (e.g., ±50, ±100, ±200, and ±300 Oe) indicate magnetization reversal. The measured magnetization M has been normalized by the saturated magnetization $M_{S}$ of the respective samples.

Owing to the random Brownian dynamics, the colloidal CMEAD-γFe_2_O_3_ nanoclusters are expected to exhibit neither remanence nor coercivity in their static magnetization at room temperature, as well as the case of hydrodynamic AOS-Fe_3_O_4_ nanostructures. By highlighting the minor hysteresis properties of the dynamic magnetization curves at 1 kHz, Figure 2 further reveals that their colloidal relaxation behaviors appear to depend on the primary size and the respective magnetic interaction of the core particles. In the case of the CMEAD-γFe_2_O_3_ ferrofluid, we observe extremely small coercivities for various field amplitudes. Under ±300 Oe, for instance, it reaches only about 0.4 Oe, much smaller than that spotted in the AOS-Fe_3_O_4_ ferrofluid. For analyzing the magnetism of the respective core particles, both ferrofluid samples were solidified by the hydrocolloid polymer (i.e., agar). We then confirm that the immobilized CMEAD-γFe_2_O_3_ nanoclusters have similar dynamic magnetization curves to those dispersed in water. In contrast, the solidified AOS-Fe_3_O_4_ ferrofluid shows an even larger hysteresis area. The coercivity of the AOS-Fe_3_O_4_ core particles increases from 0.9 Oe (under dc field) to 24 Oe (under ±300 Oe at 1 kHz).

**Figure 3:**
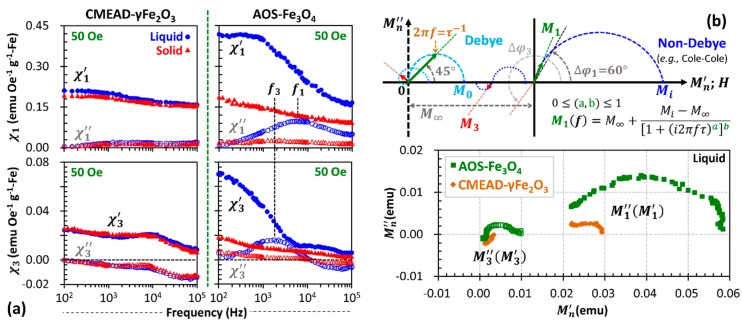
(**a**) Frequency-dependent complex magnetic susceptibilities at 50 Oe. The field-induced cluster rotation is recognized from the spectral shift of the imaginary peaks between the imaginary $\chi_{1}^{\prime \prime }$ and $\chi_{3}^{\prime \prime }$ parts (open circles), in addition the larger real $\chi_{1}^{\prime }$’ and $\chi_{3}^{\prime }$’ parts (solid circles) than those of the solidified samples (solid triangles). The imaginary parts for the solid samples (open triangles) are later attributed to the moment dynamics; (**b**) Illustrative $M_{n}^{\prime \prime }\left(M_{n}^{\prime }\right)$ plot of an empirical relaxation model with a and b fitting parameters [16]. The semicircle Cole-Cole model can be partially fitted to the $M_{1}^{\prime \prime }\left(M_{1}^{\prime }\right)$ plots of the ferrofluid samples, whereas $M_{3}^{\prime \prime }\left(M_{3}^{\prime }\right)$ appears to have unique patterns.

Referring to Figure 1a, the AOS-Fe_3_O_4_ ferrofluid may contain a mixture of superparamagnetic and ferrimagnetic magnetite nanoparticles [18]. Figure 2 further indicates that their hydrodynamic nanostructures may physically rotate under the oscillatory field to result in a hysteresis area. From Figure 3a, the effective Brownian relaxation frequency (under 50-Oe field amplitude) is observable around 6 kHz, associated with the maximum imaginary part of the fundamental susceptibility $\chi_{1}^{\prime \prime }$. This Brownian peak might be attributed to the physical relaxations of either the minor magnetite particles with diameters above 16.5 nm or the major nanoclusters with strong magnetic interactions between the composing particles (see illustration inset Figure 2). The widely distributive core sizes let them collectively behave in the liquid medium as a typical single-core structure with an effective core size [16]. Moreover, the real parts $\chi_{1}^{\prime }$ continuously decrease with frequency. The particle immobilization further results in $\chi_{1}^{\prime }$ decaying proportionally to ln(f) and the $\chi_{1}^{\prime \prime }$ being small and virtually frequency-independent [19,20,21]. Nevertheless, this typical relaxation behavior for thermally blocked magnetic nanoparticles was not the case for CMEAD-γFe_2_O_3_ nanoclusters.

### 3.2. Frequency and Field Dependences of Complex Third-Harmonic Magnetization

CMEAD-γFe_2_O_3_ nanoclusters are morphologically treated as a multicore particle system containing small superparamagnetic maghemite crystallites, in which the dipolar interaction between individual cores inside a single nanocluster is supposed to be negligible. Their fundamental susceptibility spectra appear to be identical, independent of whether the nanoclusters were suspended or immobilized (Figure 3a). Thus, all core particles within the CMEAD-γFe_2_O_3_ nanoclusters should magnetically relax via the Néel mechanism under the alternating field. There is no peak in both $\chi_{1}^{\prime \prime }\left(f\right)$ plots of the CMEAD-γFe_2_O_3_ liquid and solid samples. However, slightly larger $\chi_{1}^{\prime }$ values of the liquid sample at low frequencies might be attributed to the insignificant contribution of the Brownian dynamics. Owing to these relaxation properties, their magnetization harmonics are later found to be characteristically different from those of the AOS-Fe_3_O_4_ nanoclusters.

In principle, the equilibrium magnetization harmonics for a given sinusoidal magnetic field $H_{0}\cos\left(2\pi ft\right)$ where $H_{0}$ is the field amplitude can be mathematically derived from the Taylor series of the Langevin function in the form of Chebyshev polynomials. The Langevin equation itself ideally represents the magnetization characteristics of a single magnetic nanoparticle or a non-interacting uniform nanoparticle system. For polydispersive nanoclusters, however, one may experimentally extract the nth complex harmonic components from the time-domain magnetization responses M(t). Equations (1) and (2) further describe the real part $M_{n}^{\prime }\left(f\right)$ as

(1) $$M_{n}^{\prime }\left(f\right)=\frac{2f}{\mu_{0}}\int_{0}^{\frac{1}{f}}M\left(t\right)\cos\left(2\pi nft\right)dt,$$

and the imaginary part $M_{n}^{\prime \prime }\left(f\right)$ as

(2) $$M_{n}^{\prime \prime }\left(f\right)=\frac{2f}{\mu_{0}}\int_{0}^{\frac{1}{f}}M\left(t\right)\sin\left(2\pi nft\right)dt,$$

for one relaxation cycle at an applied frequency f, where $\mu_{0}$ is the vacuum permeability [22]. Regarding these complex magnetization harmonics $M_{n}=M_{n}^{\prime }-iM_{n}^{\prime \prime }$, we mainly discussed the third harmonic components (n=3) of the frequency- and field-dependent complex magnetic susceptibilities $\chi_{n}=M_{n}/H_{0}$ for both the real $\chi_{3}^{\prime }$ and imaginary $\chi_{3}^{\prime \prime }$ parts.

#### 3.2.1. Spectra of Third-Harmonic Susceptibility

In terms of spectral responses, the phase difference between the applied field and the resulting magnetization is one of the crucial parameters to study the complex properties of the magnetization harmonics. For instance, the third-harmonic phase difference $\Delta \varphi_{3}$ is principally three times larger than the fundamental phase difference $\Delta \varphi_{1}$ [22,23]. Hence, for an ideal Debye case with $\Delta \varphi_{1}=\tan^{-1}2\pi f\tau$ and the effective relaxation time constant τ, the maximum $\chi_{3}^{\prime \prime }$ is at the frequency that $\Delta \varphi_{3}=3\Delta \varphi_{1}$ equals π/4 rad. For the polydispersive AOS-Fe_3_O_4_ ferrofluid, accordingly, the $\chi_{3}^{\prime \prime }\left(f\right)$ plot shows such typical spectral peak difference upon $\chi_{1}^{\prime \prime }\left(f\right)$, in which it peaks at about 2 kHz, one-third of the frequency-maximizing $\chi_{1}^{\prime \prime }\left(f\right)$ plot (Figure 3a). Meanwhile, for the CMEAD-γFe_2_O_3_ ferrofluid, it is expected to have identical $\chi_{3}^{\prime }$ and $\chi_{3}^{\prime \prime }$ spectra of the solid and liquid samples due to the dominant moment dynamics. Interestingly, we found that their $\chi_{3}^{\prime \prime }$ components have negative values at low frequencies. Regarding this characteristic, the short Néel time constant of each core particle should be responsible for creating a large $\Delta \varphi_{1}$, thus $\Delta \varphi_{1}>\pi /3$ rad mathematically leads to a negative $\chi_{3}^{\prime \prime }\propto \sin3\Delta \varphi_{1}$. Being consistent with our finding, the authors of [22], furthermore, numerically identified that $\chi_{3}^{\prime }\propto \cos3\Delta \varphi_{1}$ may also be negative for this situation.

To argue that the negative $\chi_{3}^{\prime \prime }$ and positive $\chi_{3}^{\prime }$ of the CMEAD-γFe_2_O_3_ ferrofluid are not coincidental, we consider an empirical non-Debye relaxation model to evaluate the spectral magnetization responses [16,24]. As illustrated in Figure 3b, the fundamental frequency-varying magnetization $M_{1}\left(f\right)$ of the polydispersive nanoparticle system (under arbitrary field strength) settles at a nonzero value of the infinite-frequency magnetization $M_{\infty }$; $M_{\infty }=0$ for the linear relaxation response. Thus, the equilibrium (static) magnetization $M_{0}$ derived from the Langevin function may not be equivalently treated as the initial magnetization $M_{i}$ at a near-zero frequency for inhomogeneous relaxation responses. The parametric $M_{1}^{\prime \prime }\left(M_{1}^{\prime }\right)$ plot further defines $M_{\infty }$ and $M_{i}$ as the minimum and maximum values of the in-phase magnetization components $M_{1}^{\prime }$ at fundamental frequency, respectively. In the case of $M_{3}^{\prime \prime }\left(M_{3}^{\prime }\right)$ plot strongly correlated with $3\Delta \varphi_{1},$ the large $M_{\infty }$ of such non-Debye case lets $M_{3}^{\prime \prime }$ be negative for $\pi /3<\Delta \varphi_{1}<\pi /2$ rad, while $M_{3}^{\prime }$ remains positive. For the negative $M_{3}^{\prime \prime }$ attributed to the dominating moment dynamics, one may employ it as an indicator of the viscosity-related tracer immobility for a cellular MPI, in addition to the positive $M_{1}^{\prime \prime }$ as a typical estimate for the local thermal dissipation.

#### 3.2.2. Field-Dependent Third-Harmonic Susceptibility

**Figure 4:**
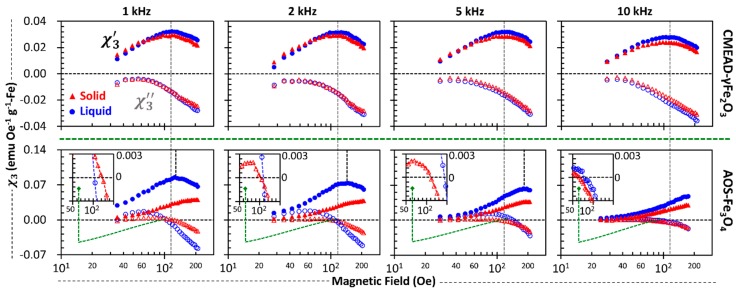
Field-dependent third-harmonic susceptibility at 1, 2, 5, and 10 kHz. The real $\chi_{3}^{\prime }$ and the imaginary $\chi_{3}^{\prime \prime }$ parts of the liquid samples (solid and open circles, respectively), as well as those of the solidified samples (solid and open triangles), distinguish the superparamagnetism of CMEAD-γFe_2_O_3_ nanoclusters from the frequency independence.

Relaxation behaviors of magnetic nanoparticles characteristically depend their frequency dependence and change with increasing field strength [24]. The field dependence of the Brownian and Néel relaxation times thus becomes an important issue to discuss the third-harmonic magnetization responses. For the CMEAD-γFe_2_O_3_ samples, Figure 4 shows that $\chi_{3}^{\prime }\left(H_{0}\right)$ peaks at relatively the same field strength regardless of frequency and only has a slight magnitude difference between the liquid and solid samples. Such a parabolic property of $\chi_{3}^{\prime }\left(H_{0}\right)$ seems to agree with the projection of the quasi-static $\chi_{3}\left(H_{0}\right)\propto H_{0}^{2}/\left(c_{0}+c_{1}H_{0}+c_{2}H_{0}^{2}+c_{3}H_{0}^{3}\right)$ derived from polynomial forms of the Langevin function for $H_{0}\geq 0$ [22], where $c_{0},$ $c_{1},$ $c_{2}$, and $c_{3}$ are constants. However, for the AOS-Fe_3_O_4_ samples, $\chi_{3}^{\prime }\left(H_{0}\right)$ is frequency-dependent and is maximized only for the liquid sample. More interestingly, their $\chi_{3}^{\prime \prime }\left(H_{0}\right)$ are negatively inverted far above 100 Oe, and the phase-inverting field amplitude appears to have a frequency dependence, as shown in Figure 4 (inset).

Being thermally blocked at low field amplitudes (i.e., having a non-zero coercivity, Figure 2), the AOS-Fe_3_O_4_ nanoparticles have Néel relaxation time constant $\tau_{N}$ that should decrease with increasing field strength. For the time required to rotate a single magnetic moment $m_{p}$, Equation (3) defines $\tau_{N}$ by emphasizing the dependences on the field strength H, particle volume $V_{m}$, anisotropy constant $k_{U}$, and temperature T; $k_{B}$ is the Boltzmann constant [24]. The pre-exponential component $\tau_{0}$ is an intrinsic time constant augmented by a random field term of the thermal fluctuations, which can be defined by Equation (4) taking the Gilbert damping factor α and electron gyromagnetic ratio γ into account [5]. Equation (3) is a general approximation of $\tau_{N}\left(H\right)$ formulated by Brown [25] in the case of low field values $H\leq 0.8\;k_{U}V_{m}m_{p}^{-1}$. Treating the Brownian time constant $\tau_{B}$ similarly as a field-dependent parameter, the shorter effective time constant above 100 Oe might create $\Delta \varphi_{1}>\pi /3$ rad to initiate the phase-inversion of the third-harmonic magnetization. In this situation, Brownian relaxation might be no longer dominant, even at frequencies below 10 kHz (Figure 4). We further believe that the negative $\chi_{3}^{\prime \prime }$ becomes a distinguishing parameter of such insignificant particle rotation. However, we still confirmed positive $\chi_{3}^{\prime \prime }$ components below 100 Oe in the case of the immobilized nanoclusters and addressed this issue to the damping behavior of the moment dynamics. Therefore, we emphasized the contribution of the α in correlation with the moment alignment within a single particle [5], instead of questioning the imperfect particle immobilization by the hydrocolloid polymer.

(3) $$\tau_{N}=\tau_{0}\exp\left[\frac{k_{U}V_{m}}{k_{B}T}{\left(1-\frac{m_{p}H}{2k_{U}V_{m}}\right)}^{2}\right]$$

(4) $$\tau_{0}=\frac{m_{p}}{\gamma k_{B}T}\frac{1+\alpha^{2}}{2\alpha }$$

### 3.3. Damping Effect on Moment Dynamics

**Figure 5:**
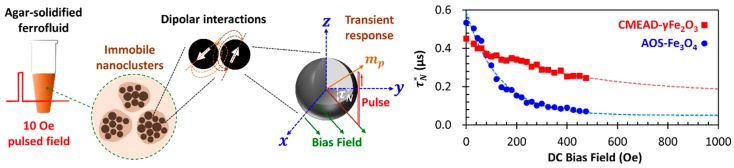
Field-dependent effective Néel time constant $\tau_{N}^{*}\left(H\right)$ of the immobilized CMEAD-γFe_2_O_3_ (solid squares) and AOS-Fe_3_O_4_ (solid circles) nanoparticles representing the damping behavior of the particle moment $m_{p}$.

Regarding how systematically the damping behavior of magnetic moments in both the CMEAD-γFe_2_O_3_ and AOS-Fe_3_O_4_ core particles contributes to their magnetization harmonics, Figure 4 highlights the maximum value of $\chi_{3}^{\prime }\left(H_{0}\right)$ and, importantly, the sign inversion of $\chi_{3}^{\prime \prime }\left(H_{0}\right)$ for the solid samples. We then investigated the magnetization decay rate of the immobilized particles by pulsating a 10-Oe magnetic field perpendicularly under a varying external dc bias field up to 450 Oe (see illustration in Figure 5); the pulse period, width, and time constant were 10, 10^−1^, and 10^−7^ s, respectively. The α was qualitatively examined from the field-dependence of $\tau_{N}$. Experimentally, $\tau_{N}$ may not satisfy Equation (3) because of the non-uniform core size distribution and dipolar interparticle interactions. The authors of [24] give an empirical approximation of the effective Néel time-constant $\tau_{N}^{*}\left(H\right)$, resembling the field-dependent Brownian time constant [22], but it appears to be limited for low-field regimes. An important issue, however, remains at high-field regimes in which $\tau_{N}^{*}$ should be proportional to the effective $\tau_{0}^{*}$ due to gyromagnetic precession [26]. Therefore, we optionally provided an empirical equation of $\tau_{N}^{*}$ by Equation (5) to include the field-independent time constant $\tau_{0}^{*}$; $a_{0}$ and $b_{0}$ are arbitrary constants.

(5) $$\tau_{N}^{*}\left(H\right)=\tau_{0}^{*}+a_{0}e^{-b_{0}H}$$

For both solid samples, $\tau_{N}^{*}$ exponentially decays with the increase in bias field, but the AOS-Fe_3_O_4_ nanoparticles exhibit a stronger field-dependence (Figure 5). The larger initial $\tau_{N}^{*}$ of the AOS-Fe_3_O_4_ nanoparticles significantly drops at H=450 Oe. We claim that this result was due to a stronger damping behavior of the respective moment dynamics. Fitting $\tau_{N}^{*}\left(H\right)$ by Equation (5), we obtained $\tau_{0}^{*}$ of 166 ns and 50.7 ns for the CMEAD-γFe_2_O_3_ and AOS-Fe_3_O_4_ nanoparticles, respectively. These values appear to overestimate those typically assumed ($\tau_{0}<1$ ns) to consider the dipolar interactions. However, less measurement data for the extrapolation may be another possible cause of high $\tau_{0}^{*}$ values. As a qualitative analysis, nevertheless, we can conclude with Equation (4) that the CMEAD-γFe_2_O_3_ nanoparticles should have a smaller α than the AOS-Fe_3_O_4_ nanoparticles, noting that they have smaller $m_{p}$ proportional to the core diameter (Figure 1a) and α<1 for both iron oxides [27,28] allows us to simplify $\left(1+\alpha^{2}\right)/2\alpha$ into 1/2α. For $\alpha \propto m_{p}/\tau_{0}^{*}$, the high damping effect on the AOS-Fe_3_O_4_ nanoparticle moments, as well as that originated from the Brownian torques on their hydrodynamic nanostructures, collectively contribute to the apparent magnetization dynamics in the case of liquid sample. Therefore, it is important to control the superparamagnetic core size of magnetic nanoclusters as the parameter contributing to $m_{p},$ α, and intrinsic dipolar interactions within the nanoclusters to achieve high complex harmonics for a functional phase-contrast MPI.

## 4. Conclusions

Magnetic interparticle interactions contribute to changing the nonlinear properties of the dynamic magnetization (i.e., complex magnetization harmonics) and highlight the field-induced particle rotation of polydispersive magnetic nanoclusters as one of the crucial factors. For superparamagnetic nanoclusters with few nanometer core particles (e.g., CMEAD-γFe_2_O_3_ nanoclusters), the negative quadrature components of the third-harmonic susceptibility were due to the fast moment dynamics creating large phase differences of the magnetization against the applied field. In contrast, for larger core particles (e.g., AOS-Fe_3_O_4_ nanoclusters), the positive quadrature components of the third-harmonic susceptibility were spectrally shifted to lower frequencies as compared with those of the fundamental susceptibility spectra. The sign inversion at higher frequencies and field amplitudes is to address the dominance of Neel relaxation over Brownian relaxation. The damping behavior of moment dynamics later becomes another important parameter to generalize the complex magnetization harmonics. We qualitatively concluded that a small damping factor is responsible for the frequency independence of the maximum in-phase components and the negative sign of quadrature components of the third-harmonic susceptibility. For such distinguishable complex components of magnetization harmonics, the magnetic nanoclusters are of the potential for MPI by suggesting an imaginary (phase) image in addition to spatially plotting the harmonic magnitudes.
